# Thrombo-pathologic features and prognosis of acute ischemic stroke patients treated with remedial stent implantation

**DOI:** 10.3389/fneur.2025.1659843

**Published:** 2025-09-16

**Authors:** Tianbo Kang, Linzhi Dai, Han Yan, Weidong Tian, Yang Wang, Caiping Wang, Yang Li, Dong Zhao

**Affiliations:** ^1^Department of Neurosurgery, The First Affiliated Hospital of Shihezi University, Xinjiang, China; ^2^Department of Neurosurgery, Baoji Central Hospital, ShanXi, China; ^3^Department of Gynecology, Affiliated Hospital of Hebei University, Hebei, China

**Keywords:** acute ischemic stroke, remedial stenting implantation, thrombophilia, no remedial stenting, thrombo-pathological features, outcome

## Abstract

**Background:**

Remedial stenting (RS) is a crucial therapy for people with acute ischemic stroke (AIS) if blood flow cannot be maintained following thrombus removal. Still, not much is known about the thrombus composition in these patients. Therefore, this study aims to compare the thrombo-pathological features of patients with AIS undergoing RS and non-remedial stenting (NRS) and to examine the correlation between thrombus composition and prognosis in patients with RS.

**Methods:**

This study included 89 patients with AIS who underwent endovascular thrombolysis. Patients were classified into RS and NRS groups based on modified thrombolysis in cerebral infarction (mTICI) scores. The primary thrombus components were identified using ICH and HE staining. The study also examined the association between thrombus components and patient prognosis.

**Results:**

The levels of neutrophil extracellular traps (NET) [0.41 (0.34, 0.61) vs. 0.36 (0.31, 0.40)], CD163 [0.31 (0.23, 0.38) vs. 0.21 (0.18, 0.23)], and C-reactive protein (CRP) [0.33 (0.26, 0.42) vs. 0.27 (0.22, 0.33)] were significantly higher in the RS group compared to the NRS group. After adjusting for other variables, both von Willebrand factor (VWF) and CD163 were associated with poor prognosis in stented patients (*P* < 0.05). The adjusted odds ratio (OR) for VWF was 2.87 × 10^6^ (95% CI: 14.44–5.71 × 10^11^), and the adjusted OR for CD163 was 4,838.64 (95% CI: 1.86–1.26 × 10^7^). Receiver operating characteristic (ROC) curve areas for VWF and CD163 in relation to poor prognosis in AIS patients 90 days after RS implantation was 0.7256 (95% CI: 0.5630–0.8870) and 0.7639 (95% CI: 0.6163–0.9115), respectively.

**Conclusions:**

Thrombi from the RS and NRS groups exhibited variations in NET, CD163, and CRP levels. In the RS group, poor patient prognosis strongly correlates with VWF and CD163 levels in the thrombi.

## 1 Introduction

Acute ischemic stroke (AIS) is the predominant cause of death and disability among adults in China, with high rates of morbidity, mortality, and long-term disability. It accounts for approximately 75%−85% of all stroke cases (1). Mechanical thrombectomy (MT) is the preferred treatment for recanalization in patients with AIS caused by major vascular occlusion (2, 3). Although MT is highly effective in achieving brain tissue reperfusion, 20%−30% of patients with AIS still experience insufficient blood flow following MT in clinical settings (4, 5). Therefore, when efficient reperfusion flow cannot be maintained following thrombus removal, intracranial remedial stenting (RS)—as a remedial therapy—offers a significant therapeutic alternative for patients with AIS.

Recent research highlights the efficacy of thrombus composition in the success of MT. Thrombi rich in erythrocytes are easier to remove because they adhere more readily to the retrieval stent (6). In contrast, fibrin-rich thrombi present a greater challenge due to their rigidity and friction, making them more difficult to dislodge and fragment (7). The physical properties of these thrombi can also cause damage to the arterial endothelium during removal, triggering an inflammatory reaction and promoting the expression of pertinent biological components. Therefore, research into the role of inflammation-related biological markers in regarding RS has garnered significant attention. These factors may indirectly affect the outcomes of RS by altering the intravascular milieu and thrombus composition. Research shows a strong correlation between thrombosis, stroke prognosis, and inflammatory markers such as von Willebrand factor (VWF), CD147, CD163, C-reactive protein (CRP), neutrophil extracellular trap (NET), and Actin (8–13). These markers can influence thrombus composition and affect reperfusion effectiveness by intensifying the local inflammatory response.

We hypothesize that inflammatory marker expression in thrombo-pathology varies among patients who undergo RS after MT, depending on the difficulty of MT in removing different thrombus components. Therefore, this study aims to investigate the relationship between thrombo-pathology and prognosis in patients with RS to reveal how inflammation-related biological factors influence thrombo-pathology characteristics and provide a theoretical basis for optimizing RS techniques. The findings could enhance reperfusion efficacy to improve the prognosis of patients with AIS.

## 2 Material and methods

### 2.1 Study design and patients

This study included 89 patients with AIS who MT at the Stroke Center of the First Affiliated Hospital of Shihezi University, China, between January 2023 and June 2024. The study protocol was approved by the ethics committee of the First Affiliated Hospital of Shihezi University, including any relevant details and written informed consent was provided by all participants and confirming that all experiments were performed in accordance with relevant guidelines and regulations. Effective recanalization was defined as an modified thrombolysis in cerebral infarction (mTICI) score of ≥2b-3 following thrombus removal (14). Based on this definition, patients with AIS were divided into two groups: RS (mTICI <2b-3) and non-remedial stenting (NRS, mTICI ≥ 2b-3). RS is defined as the inability to maintain antegrade circulation after mechanical thrombolysis owing to residual stenosis or channel reocclusion. In contrast, patients with adequate reperfusion and no severe stenosis were classified as NRS.

The inclusion criteria were as follows: (1) patients with AIS who underwent mechanical thrombus retrieval; (2) those meeting the diagnostic criteria for AIS; (3) intact thrombi collected intraoperatively, allowing for histopathological analysis; and (4) an onset-to-puncture time of ≤ 24 h.

The exclusion criteria included the following: (1) active bleeding; (2) hemorrhage or a large acute infarct (infarction covering more than one-third of the cerebral hemisphere); (3) hemorrhagic dementia, including cerebral aneurysms and cerebrovascular malformations; (4) organ failure of the heart, liver, kidneys, or other vital organs; (5) insufficient information; (6) a pre-existing Modified Rankin Scale (mRS) score >2 points; and (7) refusal to undergo pertinent imaging or endovascular therapy.

### 2.2 Data collection

We collected the following clinical information from each patient: sex, age, cerebrovascular risk factors, and pre-operative blood sugar levels, pre-operative systolic blood pressure, hemorrhagic transformations (HT), the site of thrombotic occlusion, pre-operative mTICI, and mRS scores at 90-day hospital discharge. We used the modified Rankin Scale score to assess clinical outcomes at 3 months, and defined a good clinical outcome as a score of 0–2, while a prognosis of 3–6 was deemed poor. Three months post-discharge, a follow-up telephone visit was conducted by a physician to assess the mRS scores.

### 2.3 Endovascular therapy

**Figure 1:**
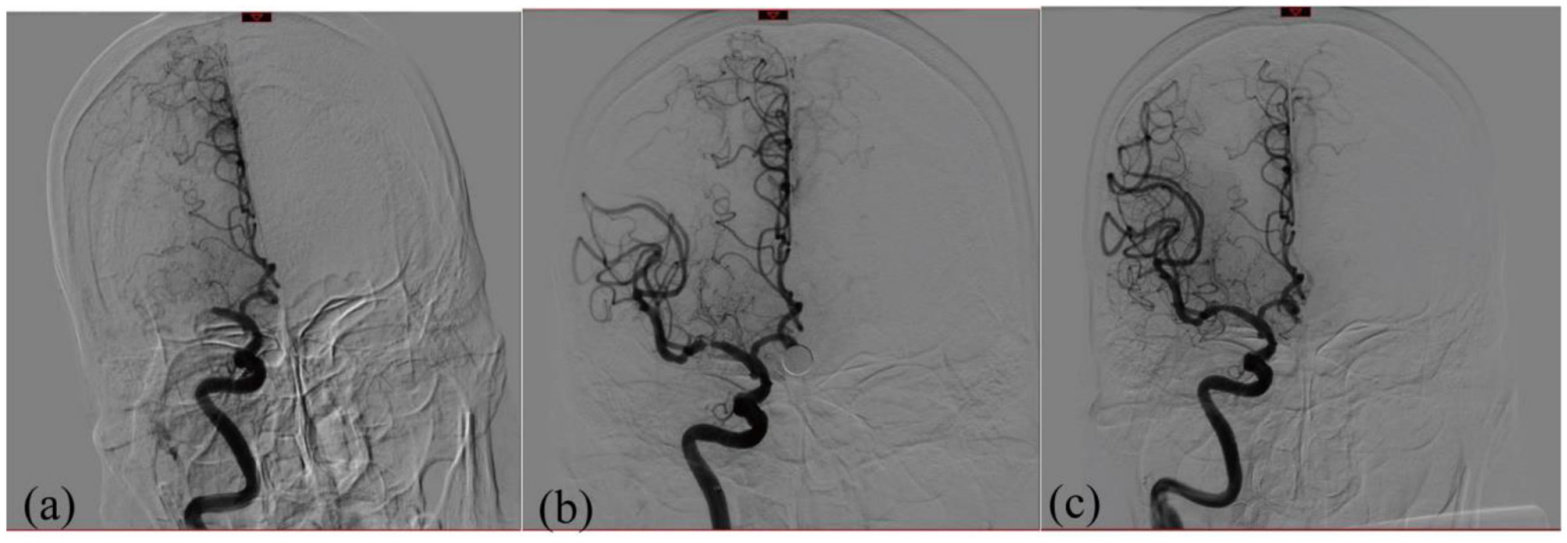
Diagram illustrating the MT recanalization procedure for acute right MCA occlusion: **(a)** patient with total MCA occlusion and loss of distal flow prior to MT; **(b)** patient with inadequate distal perfusion following MT because of MCA stenosis (mTICI <2b/3 or stenosis >70%); and **(c)** patient with full MCA restoration of distal blood flow following RS.

Using the modified Seldinger's technique, a 6F/8F catheter sheath was inserted into the femoral artery. Patients were treated under local or general anesthesia. DSA was used to promptly identify relevant vessels to evaluate collateral compensation and assess stenosis. Based on the diameter of the obstructed vessel, a Solitaire AB/FR stent was selected and positioned at the thrombus. For single-thrombus extraction, the stent and microcatheter were removed simultaneously under vacuum after a 5-min dwell period following stent release. After each mechanical embolization, vessel patency was evaluated using angiography. Based on these results, the procedure was repeated or discontinued. Generally, no more than three embolizations were performed. The procedure was concluded if the channel was effectively recanalized (mTICI ≥ 2b) following the vessel opening and observing it for 30 min (15). If effective blood flow could not be maintained after multiple thrombus removals (mTICI <2b), RS was performed (Figure 1).

### 2.4 Thrombo-pathologic staining of tissues

The thrombus was removed, frozen, and stored at −80 °C before being fixed in 4% paraformaldehyde solution and embedded in paraffin. To minimize the ice crystal effect, we used a gradual cooling method (−1 °C/min) before storage at −80 °C. All thrombus specimens were sectioned horizontally into 4.0 mm thick slices. Selected cerebral thrombus slices were subjected to immunohistochemical (IHC) staining for target proteins, including CD163 (Servicebio, GB113152, 1:700), CD147 (BOSTER, A00248-1, 1:200), Actin (Alpha-actin-2, Servicebio, GB12044, 1:1000), CRP (Proteintech, 66250-1-Ig, 1:200), VWF (Servicebio, GB11020, 1:1,000), and marker of NETs—Citrullinated Histone H3 (CitH3, 97272, CST, 1: 1,000). It is to be noted that the staining of CitH3 is primarily located in the nucleus, but it can also be found in the cytoplasm and extracellular space. Thrombus fragments were also stained for erythrocytes and fibrin/platelets (F/P) using a hematoxylin-eosin (H&E) staining solution. Micrographs of the stained specimens were obtained at 100 × magnification using an HE microscope (KIKONE100) and a Nikon camera. The mean OD for red blood cell (RBC), F/P%, CD163, CD147, Actin, CRP, VWF, and NET was determined by quantifying these components of the thrombus using ImageJ software. The 89 thrombus samples included in this study all successfully completed IHC and HE staining, with none excluded due to slide fragmentation, detachment, or poor quality. For each thrombus specimen, consecutive sections were cut, with the first section stained with H&E, and the immediately adjacent sections subjected to IHC.

### 2.5 Statistical analysis

Basic statistics are expressed as the median (interquartile range), rate, proportion, or mean ± standard deviation. The initial characteristics and differences in thrombo-pathological components between the two groups of patients with AIS were compared using the following methods. For numerical variables, if the data meet the assumptions of normality and homogeneity of variance, the *t*-test is used for two-group comparisons, and One-way ANOVA for three-group comparisons. If normality is met but homogeneity of variance is not, Welch *t*' test is used for two-group comparisons, and Welch One-way ANOVA for three-group comparisons. If the data do not meet normality, the Wilcoxon test is used for two-group comparisons, and Kruskal–Wallis for three-group comparisons. For categorical variables, when the theoretical frequency is greater than 5 and the total sample size is at least 40, the Chi-square test is applied. If the theoretical frequency is between 1 and 5 and the sample size is at least 40, Yates' correction for Chi-square is used. If the theoretical frequency is less than 1 or the sample size is smaller than 40, Fisher's exact test is recommended. Individual factors related to RS, such as thrombus biomarkers, demographics, vascular risk factors, and clinical-procedural variables, were analyzed using univariate logistic regression to determine their association with a poor mRS score at 90 days (for specific indicators, see “**Results**”). In the multivariate binary regression analysis, variables identified in the univariate analysis (*P* < 0.05) were incorporated to examine the correlation between thrombo-pathological elements in patients undergoing treatment in the stenting group and patients with a poor 90-day prognosis. Given the simultaneous analysis of multiple thrombus biomarkers, we applied the Benjamini–Hochberg method for correction during the multivariate logistic regression analysis, performed Firth penalized regression using the logistf package, used the glmnet package to validate the reliability of the results based on a larger number of variable selections, and conducted bootstrap validation using the boot and MASS packages. Statistical analyses were conducted using SPSS 27.0 and R language (version 4.2.2), with an admissible level of *P* < 0.05 for all tests. Receiver operating characteristic curves and metrics were generated using GraphPad Prism 9.

## 3 Results

### 3.1 Baseline characteristics

Based on the inclusion and exclusion criteria, 89 patients with AIS and large vessel occlusion were included in the study. We obtained 89 thrombus samples from these patients who underwent endovascular mechanical thrombolysis. Among these, 50 patients (56.2%) were classified into the NRS group and 39 (43.8%) into the RS group. Table 1 displays the baseline data for these patients. Significant differences were observed in smoking and coronary heart disease between both groups (*P* < 0.05). The etiology of stroke was classified as large artery atherosclerosis (LAA) in 44 patients (50%), cardiac origin (CE) in 33 patients (37%), and other etiologies in 12 patients (13%). A significant difference was observed in stroke etiology between the two patient groups (*P* < 0.001). Thrombus occlusion locations were as follows: internal carotid artery 24 (44%), anterior cerebral artery 6 (7%), middle cerebral artery 39 (27%), posterior cerebral artery 3 (3%), basilar artery 6 (7%), and vertebral artery 11 (12%). The distribution of thrombus occlusion sites differed significantly between both groups (*P* < 0.05). No significant differences were observed between the two groups regarding age, sex, admission National Institutes of Health Stroke Scale (NIHSS) scores, pre-operative systolic blood pressure (SBP), serum glucose (SGLU), hemorrhagic transformation (HT), and pre-operative mTICI grades (*P* > 0.05).

**Table 1:** Clinical characteristics.

**Variables**	**RS (39)**	**NRS (50)**	**Statistic**	***P*-value**
Age, Median (Q1, Q3)	61 (54, 71)	66 (59, 75)	765.50	0.084
**Gender**, ***n*** **(%)**				
Male	8 (22.5)	14 (28)	0.66	0.417
Female	31 (79.5)	36 (72)		
SBP, Median (Q1, Q3)	139 (127, 163)	135 (123, 155)	1,093.50	0.329
SGLU, Median (Q1, Q3)	7.0 (6.1, 12.1)	6.8 (5.2, 9.6)	1,065.00	0.273
NIHSS on admission, Median (Q1, Q3)	16 (12, 21)	16 (14, 22)	936.50	0.751
Diabetes, *n* (%)	16 (41)	19 (38)	0.08	0.772
Hypertension, *n* (%)	23 (59)	30 (60)	0.01	0.922
AF, *n* (%)	7 (18)	19 (38)	4.26	0.039
CHD, *n* (%)	10 (26)	19 (38)	1.52	0.217
Smoking, *n* (%)	26 (67)	22 (44)	4.53	0.033
Drinking, *n* (%)	19 (49)	18 (36)	1.46	0.227
HT, *n* (%)	8 (21)	14 (28)	0.66	0.417
**mRS, 90 days**, ***n*** **(%)**				
0–2	24 (63)	21 (42)	3.35	0.067
3–6	15 (38)	29 (58)		
**Outcome**, ***n*** **(%)**				
Survival	32 (82)	33 (66)	2.87	0.090
Death	7 (18)	17 (34)		
**Pre-operative, score on mTICI**, ***n*** **(%)**				
0	16 (41)	17 (34)		0.358
1	17 (44)	29 (58)		
2a	6 (15)	4 (8)		
**Occlusion type**, ***n*** **(%)**				
ICA	13 (33)	11 (22)		0.003
ACA	4 (10)	2 (4)		
MCA	9 (23)	30 (60)		
PCA	1 (3)	2 (4)		
BA	3 (8)	3 (6)		
VA	9 (23)	2 (4)		
**TOAST**, ***n*** **(%)**				
LAA	33 (85)	11 (22)	34.44	<0.001
CE	4 (10)	29 (58)		
Others	2 (5)	10 (20)		

NHISS, National Institutes of Health Stroke Scale; mRS, modified Rankin Score; mTICI, modified Thrombolysis in Cerebral Infarction Inventory; SBP, systolic blood pressure; SGLU, serum glucose; LAA, large artery atherosclerosis; CE, cardiac element; Others, other etiology; ICA, internal carotid artery; ACA, anterior cerebral artery; MCA. middle cerebral artery; PCA, posterior cerebral artery; BA, basilar artery; VA, vertebral artery; AF, atrial fibrillation; CHD, coronary heart disease; HT, hemorrhagic transformation.

**Figure 2:**
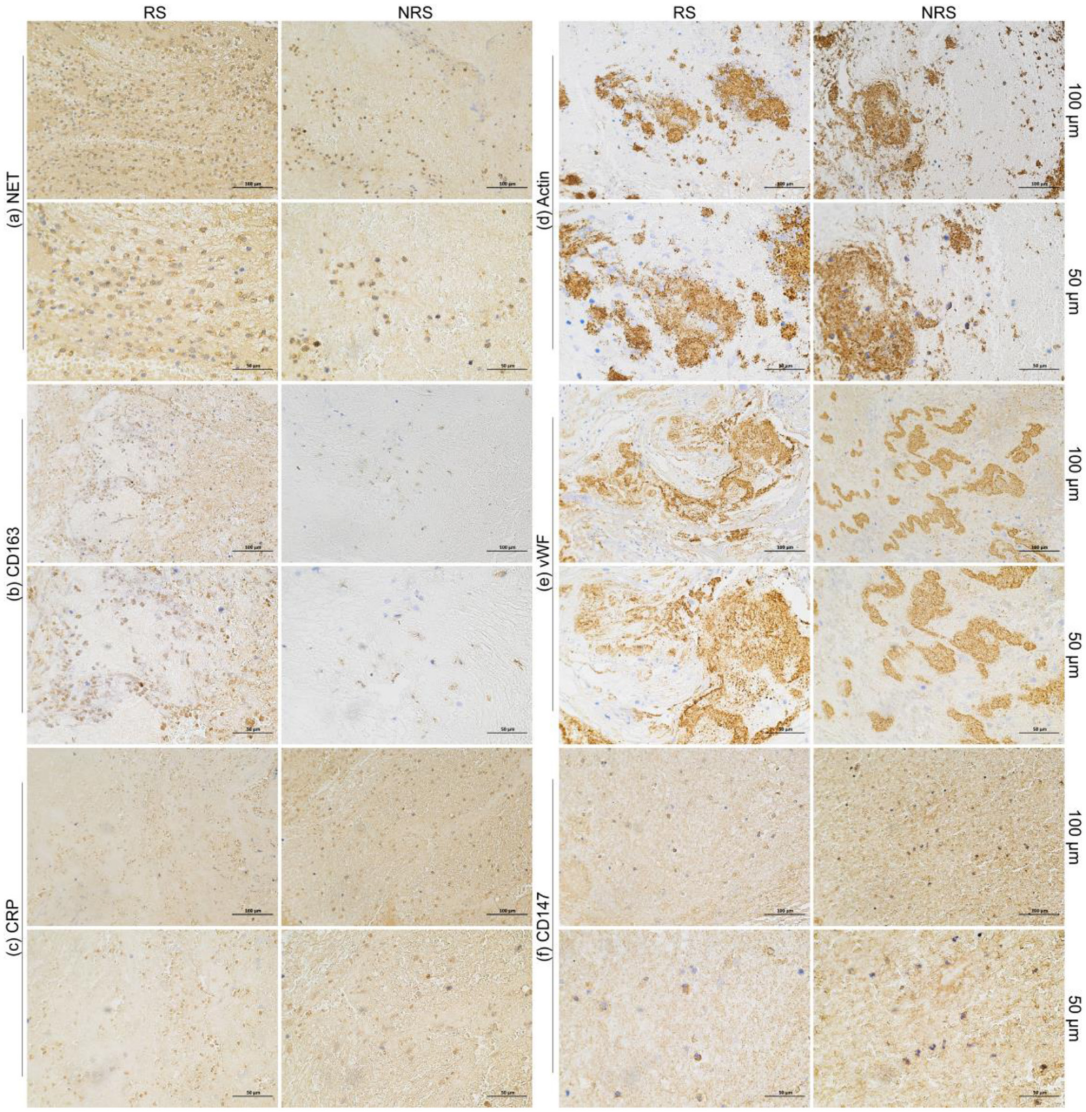
AIS patients in the RS group NRS group underwent thrombus IHC imaging (×100 magnification). The colors brown represent **(a)** NET, **(b)** CD163 (brown), **(c)** CRP (brown), **(d)** Actin (brown), **(e)** VWF (brown), and **(f)** CD147 (brown). Negative control images are not shown because of limited sample availability.

**Figure 3:**
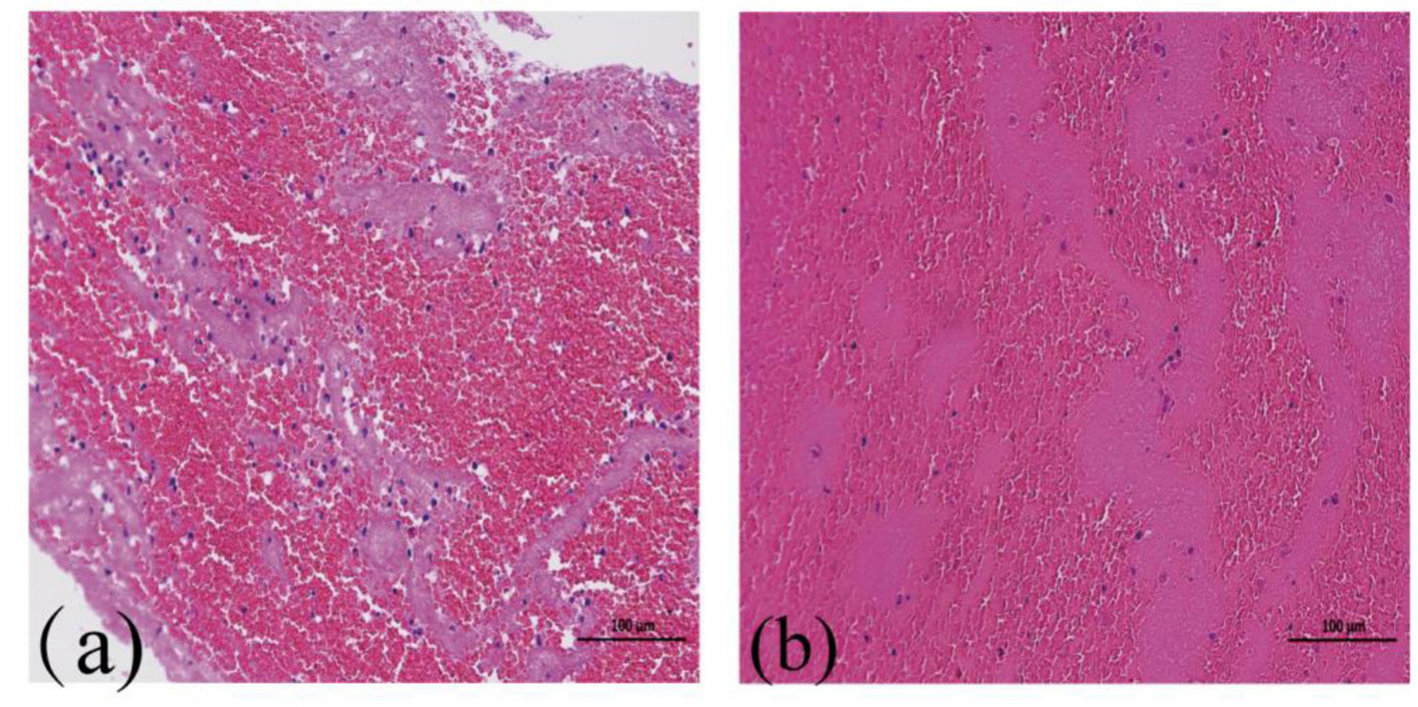
(×100 amplification) heme staining imaging of coagulation in AIS patients: **(a)** and **(b)** RBC (dark red), F/P (light red) in embolism for RS and NRS groups, correspondingly.

Examination of IHC staining images of thrombi in patients with AIS showed that key biomarkers (CD163, CRP, NET, Actin, VWF, and CD147) were uniformly distributed in patients with RS and NRS rather than being localized to specific thrombus regions. In both patient groups, Actin and VWF showed clumped, stratified, or serpentine distributions (Figure 2). RBCs were tightly clumped with platelets and fibrin in H&E-stained slices, while white blood cells were dispersed throughout these formations (Figure 3). The distribution of the major components of thrombi on H&E does not suggest a specific pattern in RS group compared to NRS group. These findings highlight key aspects of thrombus microstructure in patients with AIS and suggest that these biomarkers may play a major role in thrombosis.

### 3.2 Comparison of pathologic components of thrombus

**Figure 4:**
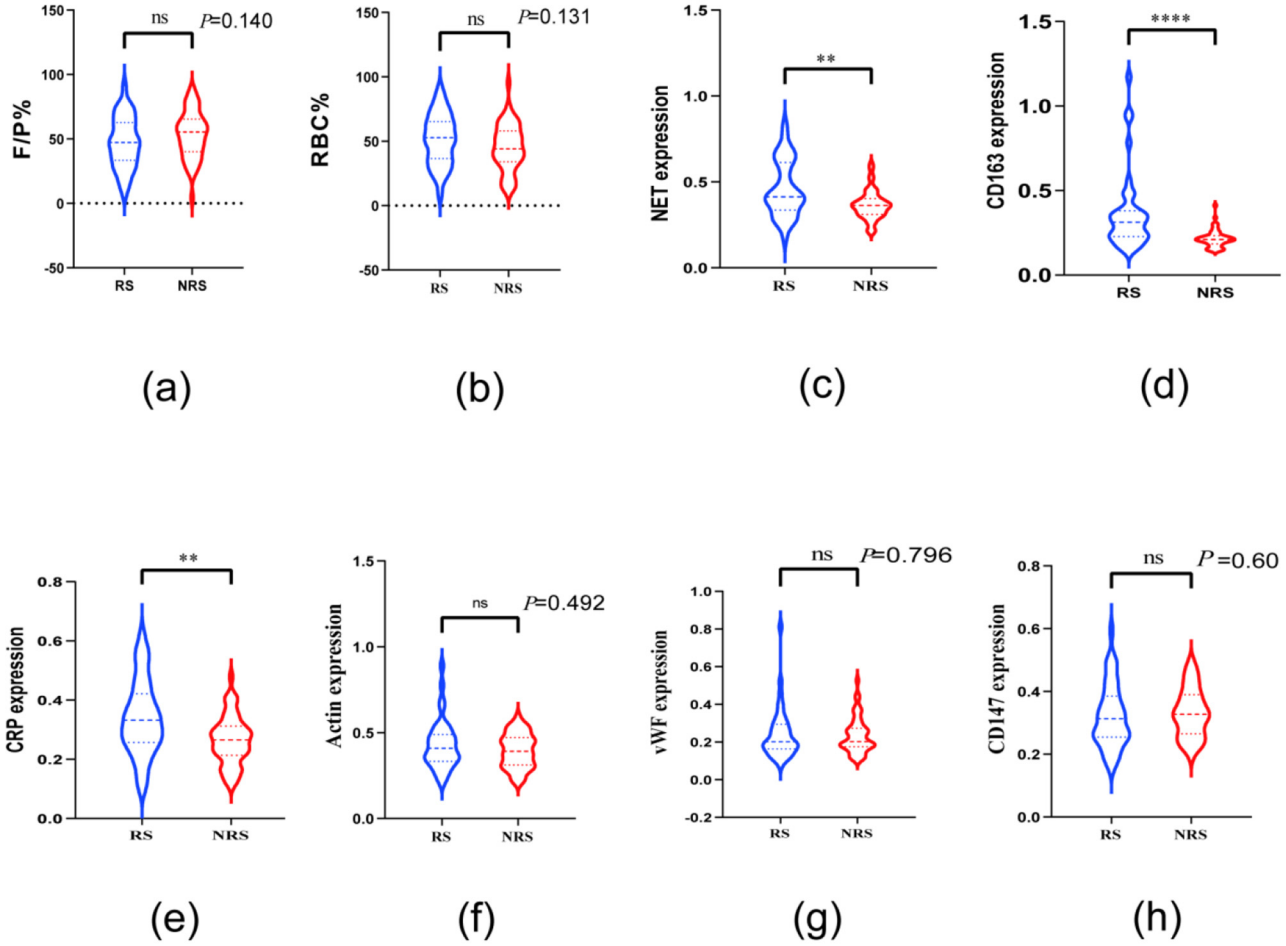
Violin plots compare the thrombopathologic characteristics of patients in the NRS and RS groups. Plots **a** and **b** reveal no differences in RBC and F/P between the two groups; plots **c, d**, and **e** reveal differences in thrombi between patients in the RS and NRS groups regarding the expression of NET, CD163, and CRP. Plots **f, g**, and **h** demonstrate CD147 in patients in the RS and NRS groups; thrombus expression of actin and vWF was not different. * denotes *P* < 0.05, ** denotes *P* < 0.01, and **** denotes *P* < 0.001.

The percentages of red blood cell (RBC) area (51.49 ± 19.77%) and F/P area (47.28 ± 19.89%) in the thrombus of patients with AIS in the RS group were similar to the RBC (45.32 ± 18.22%) and F/P areas (53.30 ± 18.17%) in the thrombus of the NRS group. No significant differences were observed in the RBC and F/P area percentages between the two patient groups (*P* > 0.05; Figures 4a, b). Patients in the RS group exhibited significantly higher levels of NET [0.41 (0.34, 0.61) vs. 0.36 (0.31, 0.40)], CD163 [0.31 (0.23, 0.38) vs. 0.21 (0.18, 0.23)], and CRP [0.33 (0.26, 0.42) vs. 0.27 (0.22, 0.33)] than those in the NRS group (Figures 4c–e). In contrast, no significant variations were observed in the expression of Actin [0.41 (0.33, 0.49) vs. 0.39 (0.31, 0.47), *P* = 0.492], VWF [0.20 (0.16, 0.29) vs. 0.20 (0.18, 0.27), *P* = 0.796], and CD147 [0.31 (0.25, 0.39) vs. 0.33 (0.27, 0.39), *P* = 0.60] between the thrombi of RS and NRS patients. No discernible variation was observed in the expression of VWF, Actin, and CD147 when comparing the thrombi of RS-treated patients with those of NRS-treated patients (Figures 4f–h).

### 3.3 Relationship between RS patient prognosis and thrombo-pathologic components

**Figure 5:**
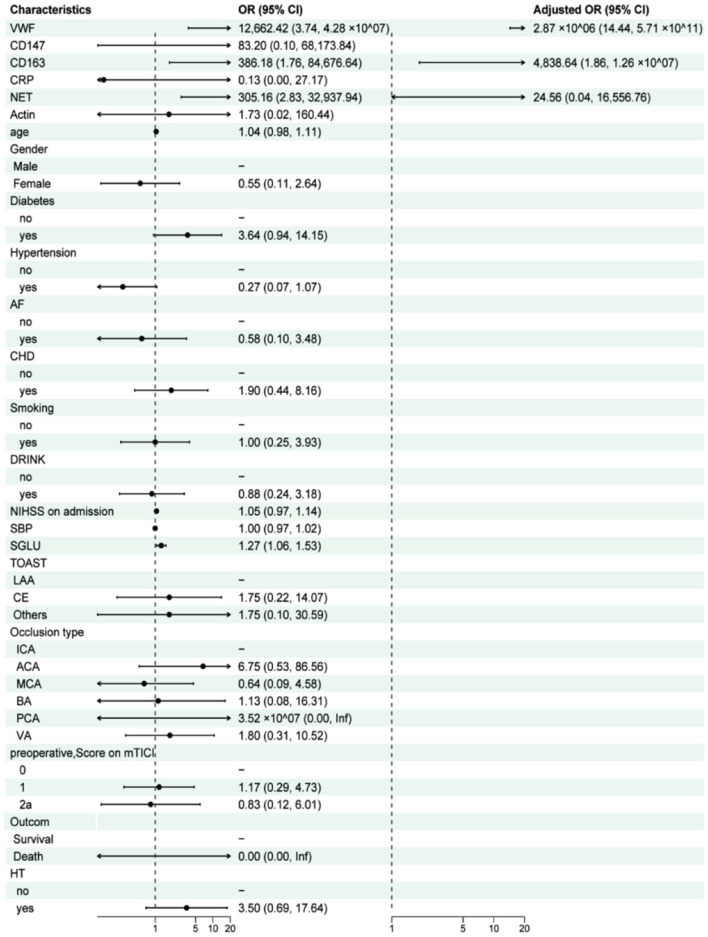
After 90 days following surgery, unifactorial and multifactorial forest plots of poor prognosis (mRS = 3–6) were seen in patients receiving remedial stent implantation for the treatment of AIS.

In this study, univariate logistic regression was employed to analyze the relationship between thrombo-pathological components of patients in the RS group (VWF, CD147, CD163, CRP, NET, and Actin) and baseline data (age, sex, cerebrovascular risk factors, admission NIHSS score, SBP, SGLU, type of TOAST, and pre-operative mTICI) with poor prognosis in patients with AIS, as indicated by mRS scores of 3–6, 90 days after surgery. In the univariate analysis, VWF (OR: 12,662.42, 95% CI: 12.27–1.51 × 10^08^), CD163 (OR: 386.18, 95% CI: 5.56–3.21 × 10^05^), NET (OR: 305.16, 95% CI: 3.90–553,818.07), and SGLU (OR: 1.27, 95% CI: 1.08, 1.57) were significantly associated with poor prognosis (*P* < 0.05). These four variables were then included in a multivariate logistic regression analysis, and after adjusting for other variables, VWF (OR: 2.87 × 10^6^, 95% CI: 14.44–5.71 × 10^11^), CD163 (OR: 4,838.64, 95% CI: 1.86–1.26 × 10^07^), and NET (OR: 24.56, 95% CI: 0.04–16,556.76) were identified as potential significant risk factors for poor mRS prognosis (Table 2, Figure 5). These findings suggest that, after adjusting for other variables, VWF and CD163 are important independent risk factors for poor 90-day prognosis in AIS patients treated with RS. It should be noted that the wide CI only serves as a directional indicator, which will be discussed in detail in the “**Discussion**” section.

**Table 2:** 90 days after the positioning of a remedial stent for AIS, patients' poor neuropsychological prognosis was determined by univariate and multivariate logistic regression analysis (mRS = 3–6).

**Characteristic**	**Univariable**					**Multivariable**				
	***N***	**Event** ***N***	**OR**	**95% CI**	***P*** **-value** ^2^	***N***	**Event** ***N***	**OR**	**95% CI**	***P*** **-value** ^a^
VWF	39	15	12,662.42	12.27, 1.51 × 10^08^	0.023^*^	39	15	2.87 × 10^06^	96.51, 7.75 × 10^12^	0.017^*^
CD147	39	15	83.20	0.12, 1.10 × 10^05^	0.196					
CD163	39	15	386.18	5.56, 3.21 × 10^05^	0.030^*^	39	15	4,838.64	12.00, 1.19 × 10^08^	0.034^*^
CRP	39	15	0.13	0.00, 24.35	0.452					
NET	39	15	305.16	3.90, 53,818.07	0.017^*^	39	15	24.56	0.04, 26,286.73	0.335
Actin	39	15	1.73	0.01, 182.09	0.813					
Age	39	15	1.04	0.98, 1.11	0.221					
**Gender**										
No	8	4	–	–						
Yes	31	11	0.55	0.11, 2.74	0.455					
**Diabetes**										
No	23	6	–	–						
Yes	16	9	3.64	0.97, 15.00	0.062					
**Hypertension**										
No	16	9	–	–						
Yes	23	6	0.27	0.07, 1.04	0.062					
**AF**										
No	32	13	–	–						
Yes	7	2	0.58	0.08, 3.19	0.556					
**CHD**										
No	29	10	–	–						
Yes	10	5	1.90	0.43, 8.44	0.388					
**Smoking**										
No	13	5	–	–						
Yes	26	10	1.00	0.26, 4.11	>0.999					
**Drink**										
No	20	8	–	–						
Yes	19	7	0.88	0.24, 3.20	0.839					
NIHSS on admission	39	15	1.05	0.97, 1.15	0.203					
SBP	39	15	1.00	0.97, 1.02	0.819					
SGLU	39	15	1.27	1.08, 1.57	0.011^*^					
**Toast**										
LAA	33	12	–	–						
CE	4	2	1.75	0.19, 16.18	0.599					
Others	2	1	1.75	0.07, 47.03	0.701					
**Occlusion type**										
ICA	13	4	–	–						
ACA	4	3	6.75	0.64, 162.80	0.142					
MCA	9	2	0.64	0.07, 4.37	0.659					
BA	3	1	1.13	0.04, 15.62	0.931					
PCA	1	1	3.52 × 10^07^	0.00, NA	0.994					
VA	9	4	1.80	0.30, 11.12	0.514					
**Pre-operative, score on mTICI**										
0	16	6	–	–						
1	17	7	1.17	0.29, 4.85	0.829					
2a	6	2	0.83	0.09, 5.78	0.857					
**HT**										
No	31	10	–	–						
Yes	8	5	3.50	0.72, 19.99	0.129					

^a*^P < 0.05; ^**^P < 0.01; ^***^P < 0.001.

OR, odds ratio; CI, confidence interval; NHISS, National Institutes of Health Stroke Scale; mRS, modified Rankin Score; mTICI, modified thrombolysis in cerebral infarction inventory; SBP, systolic blood pressure; SGLU, serum glucose; LAA, large artery atherosclerosis; CE, cardiac element; Others, other etiology; ICA, internal carotid artery; ACA, anterior cerebral artery; MCA, middle cerebral artery; PCA, posterior cerebral artery; BA, basilar artery; VA, vertebral artery; AF, atrial fibrillation; CHD, coronary heart disease; HT, hemorrhagic transformation; OR, Dominance Ratio.

**Figure 6:**
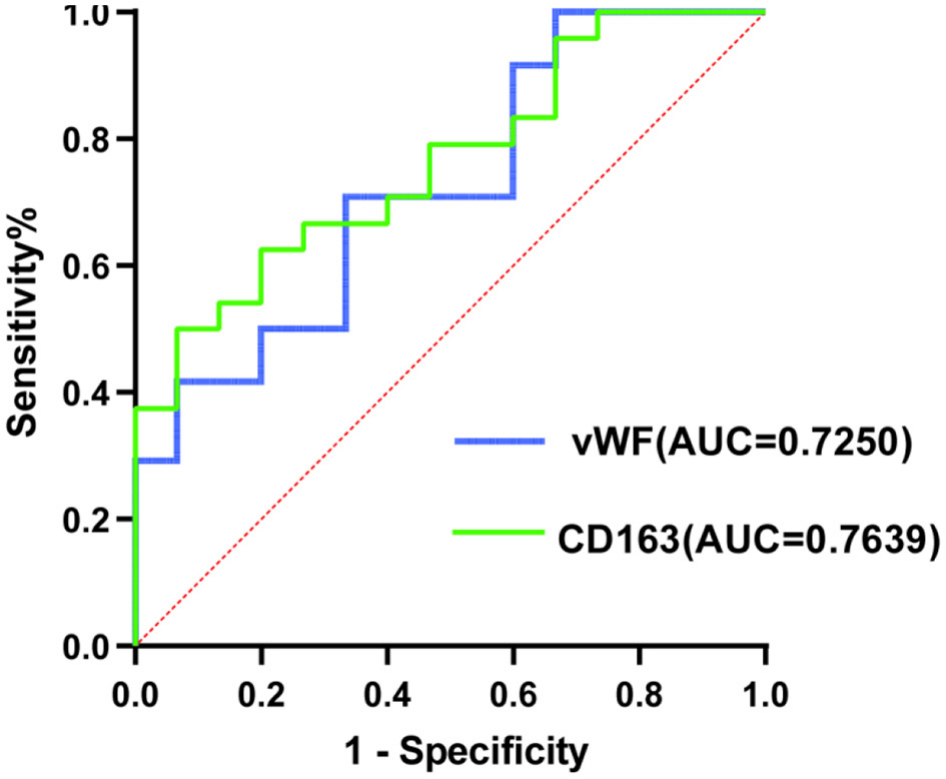
Comparing subject working curve plots of patients with poor 90-day prognosis (ROC) with insertion of a corrective stent for the therapy of embolism vWF and CD163 in patients alongside AIS.

Receiver operating characteristic plot analysis was used to assess the sensitivity and specificity of VWF and CD163 in thrombi from patients with AIS treated with RS implantation in relation to their prognosis at 90 days post-operatively. The findings reveal that the VWF and CD163 curve regions associated with poor prognosis at 90 days post-surgery were 0.7256 (95% Cl.0.5630–0.8870) and 0.7639 (95% Cl.0.6163–0.9115), respectively (Figure 6).

## 4 Discussion

Our findings are as follows: (1) VWF, CD147, Actin, white blood cells, and F/P content showed similar expression in the thrombi of both patient groups. However, thrombi from patients with RS exhibited higher levels of CD163, CRP, and NET than those from patients with NRS. (2) In patients with RS, the thrombo-pathological factors VWF and CD163 independently predict a poor prognosis at 90 days (mRS: 3–6).

The evidence suggests that RS occurs following thrombus extraction in patients with AIS, primarily owing to thrombus diversity (including stiffness and friction) and atherosclerosis-induced stenosis, among other factors (16). These variables can lead to endothelial injury and residual thrombus, causing hemodynamic anomalies, inflammatory reactions, and impaired blood flow post-thrombus removal. In contrast to patients with NRS, thrombi in patients with RS can be further exacerbated by hemodynamic alterations, inflammatory responses, and associated biological variables. Our study shows that thrombi from patients with RS exhibit higher NETs, CD163, and CRP levels, along with greater cellular content, than those of patients with NRS. Additionally, CD163 and VWF were poor prognostic markers in patients with RS. These findings suggest that in patients undergoing RS following thrombus ablation, biomarkers such as inflammatory variables may be important in thrombosis and reperfusion processes.

This study reveals that thrombus NET expression is higher in RS patients compared to NRS patients. NETs are crucial components of thrombi in ischemic stroke and contribute to the pathogenesis of AIS. Research indicates that NETs in the thrombus of patients with AIS resist thrombolysis and prevent the recanalization of blocked arteries (17). First, NETs, with their specialized mesh fiber structure, trap and encapsulate red blood cells, platelets, and other components (18). Furthermore, NET DNA and other components alter the fibrin structure, enhancing the physical properties of the thrombus and limiting its total removal (19). A study found a dense fibrin network containing VWF and NETs in the thrombus of AIS patients, which provides an adhesive scaffold for red blood cells, platelets, fibrin, and coagulation factors, making the thrombus difficult to dissolve. The researcher suggested targeting multiple thrombus components in thrombolytic therapy to benefit patients clinically (20). Residual NETs in the thrombus can trigger platelet activation and local inflammatory response, increasing the risk of re-thrombosis and impairing blood flow continuity and stability following thrombus removal. Second, a high neutrophil count correlates closely with disease severity and is a significant predictor of AIS (21). Neutrophils in patients with AIS release substantial amounts of NETs, which can directly cause endothelial dysfunction by activating and damaging endothelial cells, attracting inflammatory cells involved in atherosclerotic plaque formation, and leading to localized cerebral artery stenosis. Other studies have shown that NETs are involved in the composition of all AIS thrombi, especially in their outer layers. Quantitative measurement of thrombus NETs content was not associated with clinical outcomes or AIS pathogenesis, but was significantly correlated with the procedure length of endovascular therapy and the number of device passes (22). Consequently, we examine how these elements interact. NETs not only hinder blood flow from achieving effective reperfusion after thrombus retrieval but also enhance the physical properties of the thrombus and promote inflammation and vasculopathy.

Studies show that CRP is a significant potential biomarker for assessing the prognosis of patients with AIS (23, 24). CRP is commonly used to assess the pathologic inflammatory response and has been extensively studied in relation to thrombosis linked to atherosclerosis. This study suggests a direct correlation between high CRP levels in the thrombi of patients with RS and their impaired ability to restore blood flow after thrombus removal. CRP contributes to oxidative stress and the generation of reactive oxygen species (ROS), among other roles, in the atherosclerotic process (25). These ROS can oxidize low-density lipoprotein cholesterol, producing oxidized low-density lipoprotein, an essential mediator in the development of atherosclerotic plaques (26). Recurrent thrombus removal may lead to endothelial damage and plaque rupture in patients with AIS, initiating a vicious cycle that exacerbates endothelial injury, increases vascular permeability, increases CRP production, and activates inflammatory responses. This cycle enhances the possibility of blood clot formation and adhesion to injured arterial walls, thereby obstructing blood flow. Additionally, CRP activates the complement system, which promotes thrombus formation (25). Activation of the complement system promotes platelet aggregation, supports the formation of a stable thrombus structure, and converts fibrinogen to fibrin. CRP also significantly stimulates the migration and proliferation of vascular smooth muscle cells. These cells serve as bridges during coagulation, enhancing thrombus durability and reducing the likelihood of complete dissolution. Based on the cited studies, CRP expression in thrombi is likely higher in patients with RS than in those with NRS.

Research indicates that CD163 can serve as a biomarker for various diseases, including infections, autoimmune diseases, cancers, and nervous system abnormalities (27). CD163 is a significant molecular marker on the surface of macrophages, binding to erythrocytes and their metabolites to facilitate their removal. Its elevated expression in tissues often indicates a specific adaption mechanism to the inflammatory response (28). In patients with AIS, CD163 is necessary for the local immune response involving brain microglia and circulating monocytes (29). The pathogenesis of AIS is correlated with the role of CD163 in stimulating the anti-inflammatory response of monocytes (30). Therefore, we hypothesize that in patients with AIS undergoing RS, CD163 is predominantly expressed in monocytes and macrophages. These cells can adhere to the arterial wall during thrombosis and release inflammatory mediators such as interleukin-6 and tumor necrosis factor-α. These inflammatory agents exacerbate endothelial damage and contribute to the atherosclerotic process, which narrows the arterial lumen and thickens the arterial wall, thereby impeding effective blood circulation and reperfusion following thrombus removal.

In this study, where we examined thrombi in patients with RS and NRS, minimal variation was observed in the expression of key biomarkers and their components, such as VWF, CD147, actin, RBC, and F/P, between the two groups. The reasons for selecting above biomarkers for detection are as follows. High levels of actin in thrombi enhance the red blood cell-fibrin scaffold, making it difficult to completely remove the thrombus during the first thrombectomy, which may require stent rescue to improve treatment outcomes (4); CD147 upregulates MMP-9, disrupting the blood-brain barrier and amplifying local inflammation, and has been shown to be closely associated with post-operative reperfusion injury and hemorrhagic transformation (8); VWF, released in large quantities after endothelial denudation, rapidly mediates platelet adhesion, triggering early reocclusion (31). The above indicators are crucial for the success rate of stent implantation, thrombosis formation, and vascular stability. This study shows that the proportion of LAA is higher in the RS group, while the proportion of CE is higher in the NRS group. Previous studies have indicated that there is no significant difference in RBC and F/P expression in thrombi from CE-type and LAA-type stroke patients (32). The infiltration of neutrophils and the stimulation of oxidized low-density lipoprotein within atherosclerotic plaques can induce the formation of NETs. Repeated mechanical traction or residual stenosis during thrombectomy may also cause persistent endothelial damage, triggering neutrophils to release large amounts of NETs. NETs, in turn, can enlarge the thrombus volume by capturing red blood cells and platelets, but they do not alter the relative proportions of F/P and RBC. This could be the potential reason for the lack of significant differences in F/P and RBC ratios between the two groups, while there is a significant difference in NETs content. The specific mechanisms involved in this process need to be further investigated in future studies.

These preliminary findings suggest that endothelial damage and inflammation are crucial in AIS development, with high CD163 expression levels potentially indicating a poorer prognosis. Jiang et al. (27) found a strong connection between CD163 levels and poor outcomes in patients with AIS based on embolism studies involving 25 patients with LAA-type strokes and 42 CE-type strokes. Greco et al. (33) categorized human monocytes into CD16+ and CD14+ subtypes, revealing a significant correlation between stroke severity and disability and CD163 levels in CD16+ monocytes in patients with ischemic stroke. This is in line with the findings of our study in patients with AIS undergoing RS treatment, where we hypothesize that repeated thrombus removal contributes to endothelial damage. This damage increases vascular permeability, which is stimulated by CD163, leading to inflammatory mediator release and activation of VEGF-A/VEGFFR2 signaling by macrophages through hemoglobin clearance (34). Such changes compromise the integrity of the blood-brain barrier, increasing the risk of hemorrhagic transformation and malignant cerebral edema, thereby worsening patient prognosis.

This study revealed a correlation between elevated levels of VWF and both poor prognosis and stroke severity (35). VWF, a glycoprotein produced by vascular cells, plays a crucial role in blood coagulation and platelet adhesion. Abnormally high VWF levels are associated with thrombosis, inflammation, and endothelial cell damage (24). Given that patients with AIS undergoing RS can experience stenosis after thrombus removal, high shear rates in such cases may cause VWF to stimulate inflammation by binding to GPIb on platelets, thereby forming a VWF-GPIbα axis (36). This leads to leukocyte recruitment and edema, triggering an allergic cascade and reducing the efficiency of intravascular blood flow perfusion in the cerebral infarction region. Moreover, thrombus removal damages endothelial cells, causing a significant spillover of VWF. This initiates the thrombo-inflammatory cycle, which exacerbates clinical impairment in patients with AIS and is connected to ischemia, reperfusion (I/R) injury, and the post-ischemic inflammatory response (37). Therefore, we conclude that these factors collectively represent a positive association between VWF and both stroke severity and poor prognosis in patients with AIS.

However, analyzing the thrombo-pathological characteristics of patients with AIS who received RS vs. NRS showed significant differences in the expression of biomarkers associated with AIS and thrombosis, such as CD163, NET, and CRP, between the two patient groups. While VWF levels were similar across both groups, poor prognosis in patients with RS was strongly associated with elevated VWF and CD163 levels. This study has some limitations. It is a retrospective, single-center study, which introduces potential selection bias. The RS group only included 39 cases, with 15 cases showing poor prognosis. The events-per-variable (EPV) ratio was far below the commonly used EPV ≥10 rule for logistic regression. A low EPV can lead to instability in maximum likelihood estimates, manifesting as extreme point estimates (with OR often >1,000) and confidence intervals spanning multiple orders of magnitude, we therefore, attempted the following statistical methods to validate the reliability of the results. Due to the inclusion of too many variables, we attempted variable selection using Lasso regression. Based on Supplementary Table S1, we found that the selected variables still retained VWF, CD163, and NET, which were reported in the “**Results**” section. This suggests that despite the small EPV, our results are meaningful. Wide confidence intervals do not indicate that the “true effect is large,” but rather reflect the statistical uncertainty due to the small sample size. Therefore, the study may carry an elevated risk of both type I (false-positive) and type II (false-negative) errors, and all ORs should be considered as “directional indicators” rather than precise clinical effects. Here, we used the logistf package (38) to perform Firth penalized regression to attempt to eliminate extreme estimates caused by small sample sizes. After shrinkage, the OR for VWF, CD163, and NET are much smaller than the OR reported in the manuscript, but it still remains statistically significant. Thus, although the OR is large, our results remain meaningful after shrinkage (Supplementary Figure S1). We also used bootstrap (1,000 resamplings) to validate the stability of the OR results from multivariate logistic regression. The results showed that the bootstrap OR trends for VWF (1.15 × 10^11^), CD163 (1.17 × 10^10^), and NET (2.79 × 104) were consistent with those reported in the results, and all fell within the confidence intervals (Supplementary Table S2), suggesting that the trends identified in our analysis are relatively reliable. We then supplemented with receiver operating characteristic (ROC) - area under the curve (AUC) to make the predictions more robust. It is important to note that based on the above “directional results,” the ROC curve showed that both VWF (AUC 0.7256) and CD163 (AUC 0.7639) fall within the 0.7–0.8 range, indicating moderate individual predictive ability, thus VWF/CD163 can serve as a “red flag” indicator, alerting the surgeon to develop a rigorous post-operative monitoring or enhanced antiplatelet strategy in advance, rather than directly deciding “whether to place a stent.” There are also some other limitations in this study. In the multivariate logistic regression analysis, only variables with *P* < 0.05 in univariate screening were included; confounding factors such as age, sex, and comorbidities were not included in the analysis, which may leave residual confounding. In addition, timely blood specimens and detailed clinical data, such as the number and duration of procedures, were not obtained, and data from only three views of a single thrombus section were used in this study, which may not fully represent the thrombus. Moreover, owing to the scarcity of residual thrombus material, dedicated sections for isotype-matched negative controls could not be allocated. Given the small sample size and potential biases in data analysis, further multicenter studies with larger sample sizes and stratification are needed to identify and validate the independent predictive value of more biomarkers, while minimizing confounding factors as much as possible.

## 5 Conclusions

Patients receiving NRS did not exhibit a statistically significant difference in Actin, VWF, CD147, RBC, and F/P levels in their thrombi compared to those receiving RS treatment. In contrast, thrombi from patients undergoing RS exhibited significantly higher levels of CD163, CRP, and NET. Furthermore, elevated VWF and CD163 levels in thrombi from patients with RS were linked to poor prognosis for 90 days. These findings suggest that relevant bleeding biomarkers may be valuable targets for future treatments and offer significant prognostic insights for managing RS in patients with AIS.

## Data Availability

The original contributions presented in the study are included in the article/Supplementary material, further inquiries can be directed to the corresponding author.
