# Supplementary material for: Thrombo-pathologic features and prognosis of acute ischemic stroke patients treated with remedial stent implantation
Source: Front Neurol. 2025 Sep 16;16:1659843. doi: 10.3389/fneur.2025.1659843 (PMC12479334; doi:10.3389/fneur.2025.1659843)
Supplement: Supplementary file 1 [file Supplementary_file_1.docx]

1. Due to the inclusion of too many variables, we attempted variable selection using Lasso regression. Based on Table.S1, we found that the selected variables still retained VWF, CD163, and NET, which were reported in the "Results" section. This suggests that despite the small EPV, our results are meaningful.

Table.S1 Validation of variable selection in logistic regression based on Lasso regression.

| variable | lambda.min |
| --- | --- |
| (Intercept) | -7.599500082 |
| VWF | 8.149460463 |
| CD163 | 3.292971122 |
| CRP | -1.869913147 |
| NET | 4.73824154 |
| BP | -0.135703228 |
| DRINK | -0.114265852 |
| SGLU | 0.224173494 |
| HT | 1.625937366 |

We also used bootstrap (1,000 resamplings) to validate the stability of the OR results from multivariate logistic regression. The results showed that the bootstrap OR trends for VWF (1.15 × 10¹¹), CD163 (1.17 × 10¹⁰), and NET (2.79 × 10⁴) were consistent with those reported in the results, and all fell within the confidence intervals (Table.S2), suggesting that the trends identified in our analysis are relatively reliable.

Table.S2 Validation of the stability of the OR results from multivariate logistic regression based on bootstrap.

| Variable | OR_point | OR_boot | CI_lower | CI_upper |
| --- | --- | --- | --- | --- |
| VWF | 12662.4185 | 1.145191e+11 | 45.090765 | 439630109.3 |
| CD163 | 386.1782 | 1.166197e+10 | 19.153998 | 11750655.5 |
| NET | 305.1575 | 2.792201e+04 | 5.769834 | 140740.9 |

the R code is as follows：

# 安装并加载必需包

# install.packages(c("readxl", "boot", "MASS")) # 首次运行请取消注释

library(readxl)

library(boot)

library(MASS)

# 读取数据

dat <- read_excel("分析数据/分析数据.xlsx")

# 定义 Logistic 回归函数

logit_or <- function(data, indices, predictor) {

d <- data[indices, ]

fit <- glm(outcome ~ d[[predictor]], family = binomial, data = d)

exp(coef(fit))[2] # 返回 OR

}

# Bootstrap 设置

set.seed(2024)

B <- 1000 # bootstrap 次数

vars <- c("VWF", "CD163", "NET")

boot_res <- lapply(vars, function(v) {

boot.obj <- boot(dat, logit_or, R = B, predictor = v)

ci <- boot.ci(boot.obj, type = "perc")$percent[4:5] # 百分位 CI

data.frame(Variable = v,

OR_point = exp(coef(glm(outcome ~ ., dat[, c("outcome", v)], family = binomial))[2]),

OR_boot = mean(boot.obj$t),

CI_lower = ci[1],

CI_upper = ci[2])

})

# 合并结果并打印

boot_df <- do.call(rbind, boot_res)

print(boot_df)

2. We performed Firth penalized regression using the logistf package, and the R code is as follows：

library(logistf)

fit_firth <- logistf(outcome ~ VWF + CD163 + NET, data = df)

summary(fit_firth)

exp(cbind(OR = coef(fit_firth), confint(fit_firth)))

The results are as follows:


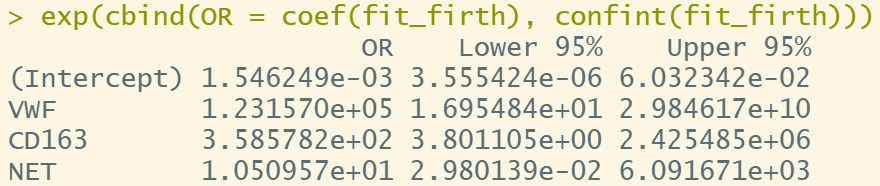


Fig.S1 The OR and 95% CI of VWF, CD163, and NET after Firth penalized regression shrinkage in multivariate analysis.

Therefore, after shrinkage, the OR for VWF, CD163, and NET are much smaller than the OR reported in the manuscript, but it still remains statistically significant. Thus, although the OR is large, our results remain meaningful after shrinkage.
